# Capturing the patient voice: implementing patient-reported outcomes across the health system

**DOI:** 10.1007/s11136-019-02320-8

**Published:** 2019-10-12

**Authors:** Elizabeth Austin, Cynthia LeRouge, Andrea L. Hartzler, Courtney Segal, Danielle C. Lavallee

**Affiliations:** 1 Surgical Outcomes Research Center, University of Washington, 1107 NE 45th Street, Suite 502, Box 354808, Seattle, WA 98105 USA; 2 Information Systems and Business Analytics, Florida International University, Miami, FL USA; 3 Department of Health Services, University of Washington, Seattle, WA USA; 4 Department of Biomedical Informatics and Medical Education, University of Washington, Seattle, WA USA

**Keywords:** Patient-reported outcomes, Patient engagement, Depression, Care transformation, Action research, Implementation

## Abstract

**Purpose:**

Supporting the capture and use of patient-reported outcomes (PROs) at the point-of-care enriches information about important clinical and quality of life outcomes. Yet the ability to scale PROs across healthcare systems has been limited by knowledge gaps around how to manage the diversity of PRO uses and leverage health information technology. In this study, we report learnings and practice insights from UW Medicine’s practice transformation efforts to incorporate patient voice into multiple areas of care.

**Methods:**

Using a participatory, action research approach, we engaged with UW Medicine clinical and administrative stakeholders experienced with PRO implementation to inventory PRO implementations across the health system, characterize common clinical uses for PROs, and develop recommendations for system-wide governance and implementation of PROs.

**Results:**

We identified a wide breadth of PRO implementations (*n* = 14) in practice and found that nearly half (47%) of employed PRO measures captured shared clinical domains (e.g., depression). We developed three vignettes (use cases) that illustrate how users interact with PROs, characterize common ways PRO implementations support clinical care across the health system (1) *Preventive care,* (2) *Chronic/Specialty care*, and (3) *Surgical/Interventional care)*, and elucidate opportunities to enhance efficient PRO implementations through system-level standards and governance.

**Conclusions:**

Practice transformation efforts increasingly require integration of the patient voice into clinical care, often through the use of PROs. Learnings from our work highlight the importance of proactively considering how PROs will be used across the layers of healthcare organizations to optimize the design and governance of PROs.

**Electronic supplementary material:**

The online version of this article (10.1007/s11136-019-02320-8) contains supplementary material, which is available to authorized users.

## Introduction

Transformation to higher value, lower cost care is dependent on the ability of healthcare systems to leverage data that drive improvements in patient care and population health [1, 2]. Targeted engagement with patients in care delivery and coordination further supports the aims of patient- and family-centered care [3, 4]. In particular, supporting the capture and use of patient-reported data enriches information about important outcomes and ensures the patient voice is central to care delivery.

Patient-reported outcomes (PROs) provide standardized assessments for collecting data directly from patients about health status or experience with a health condition [5]. When integrated into care, PROs can facilitate patient–provider communication, detection and management of health conditions, and improve patient satisfaction [6]. Examples include reporting of treatment-related side effects among patients undergoing chemotherapy [7], improved recognition of depression among children with diabetes [8], and the ability to track and trend changes in pain and function following surgical interventions [9]. The utility of PROs depends on rigorous quality in the collection and stewardship of patient-reported data to ensure timely access of accurate information [10, 11]. While conventionally collected using paper-based workflows, electronic health records (EHR) enable the capture and scoring of PROs in real time. Coupling technology with widespread use of PROs across care settings can support efficiencies in care delivery while augmenting downstream quality improvement, population health, and payment policy [12].

In 2016, University of Washington (UW) Medicine, a four-hospital health system in the Seattle metropolitan area, launched a system-wide practice transformation initiative to promote evidence-based, patient-centered care. The objectives of this initiative included the use of standard care pathways, increased engagement with patients outside the care setting, and greater inclusion of patient voice in clinical documentation and decision-making. For example, UW Medicine developed an EHR-supported care pathway to guide primary and specialty care teams in the management of depression, including plans for routine capture of depression PROs [13], such as the Patient Health Questionnaire (PHQ) [14]. While the goals for depression management are consistent across clinical settings, the proposed implementation of pathway recommendations and the use of PROs vary significantly depending on clinical context. These issues are further complicated when workflows for PRO-based depression screening are layered over workflows for depression management, as the processes for PRO data review differ across settings and purpose.

Early implementations of PROs at UW Medicine reflect clinical use in narrow settings of care (e.g., spine surgery [15], HIV care [16], and cancer treatment [17])—not scaled across the healthcare system. Although these efforts provided valuable insights about how PROs support patient care in specific settings, these single scenarios limited our understanding of how to optimally scale electronic PRO implementation, balancing the needs of diverse care settings and patient populations. System standardization uses a global approach to leverage economies of scale, limit time and cost for broad-scale implementations, and promote rapid learning across settings and groups [18, 19]. However, a “one size fits all” approach can compromise effective localized implementation and the potential benefits PROs bring to clinical care. Prior research highlights the potential value in identifying a limited number of clinical scenarios (i.e., “use cases”) that reflect important commonalities and differences across settings to guide governance structures and implementation decisions [20]. Use cases are often presented as vignettes illustrating how users interact with a system or process (e.g., use of PROs) [21, 22]. Use cases help clarify value propositions held by stakeholders, key features of implementations, training requirements, change management practices, and barriers to adoption [23, 24]. Thus, use cases offer a valuable tool for examining how to scale PRO implementation across health systems.

The present study was led by a multidisciplinary research team of experts in user-centered design, biomedical informatics, systems engineering, health services research, and national guideline development for PROs in clinical practice [25]. We collaborated with UW Medicine stakeholders to describe how PROs are currently used across the health system, and identify common use cases to serve as the blueprint for the design of system-wide PRO implementation strategies.

## Methods

Using a participatory action research approach designed for healthcare contexts [26], we engaged stakeholders using a combination of electronic questionnaires and in-person interviews to inventory current PRO implementations across UW Medicine, characterize common use cases for PROs, and develop recommendations for a system-wide implementation strategy. Action research is advantageous for studying healthcare settings where clinical stakeholders have deep contextual knowledge about their environments and where researchers benefit from actively participating in the topic being studied [27–29]. Through cycles of planning (identifying issues, research questions, and inquiry methods), acting (gathering data), observing (health system project activities and meetings), and reflecting (analyzing data and communicating) [30], the research team informed UW Medicine clinical practice transformation and PRO stakeholder communities as they strategized about future system-wide PRO implementations. UW Medicine PRO stakeholders included the UW PROs Governance Committee members—a body of diverse clinical, administrative, informatics, and stakeholders charged with providing guidance on PRO implementation to the UW Medicine health system—as well as a broader learning community of stakeholders with known interest or experience in PRO implementation for clinical care. Engaging in iterative reflection with stakeholders within the UW Medicine environment where change was occurring allowed us to align findings with real-world practice.

We summarize our action research approach across three distinct phases: inventorying PRO use, characterizing common uses of PROs in clinical care, and contextualizing use cases using the example of the PHQ (Table 1). Each phase involved continuous cycles of planning, acting, observing, and reflecting. Data collection and analysis activities were approved by the UW Institutional Review Board (IRB), and informed consent was obtained for appropriate activities (e.g., interviews) in accordance with UW IRB policies.

**Table 1:** Action Research approach

Phase	Purpose and corresponding action research cycle [30]	Data collection and analysis	Results/outputs
Phase 1: Inventorying current PRO use	Information gathering about diversity of PROs used and characteristics of PRO implementations across health system (*planning, acting*)	Electronic questionnaire analyzed through descriptive statistics; open-ended responses and supporting PRO-related documentation (i.e., materials about PRO implementations provided by stakeholders) explored through content analysis	Inventory of the breadth of PRO implementations in which stakeholders used PROs
Phase 2: Characterizing common uses of PROs in clinical care	Information gathering to understand the context and workflow of PROs in various clinical settings (*acting*, *observing*) Reflection on inventory and interviews to identify common use cases (*reflecting*)	Semi-structured interviews evaluated through content analysis and member checking with stakeholders	Detailed description of how PROs are used in care delivery across diverse settings Identification of common use cases for PROs in clinical care
Phase 3: Contextualizing common use cases for PROs using PHQ	Apply proposed use cases to PHQ through collaborative interpretation with stakeholders (*observing, reflecting*) Illustrate clinical scenarios to guide system-level design and governance (*planning*)	Model patterns of PRO capture and reporting across practice settings Solicit iterative stakeholder feedback on iterative model evaluated through content analysis and member checking	Validation of PRO use cases recommended for system-wide PRO implementation strategy Summarization of learnings about the clinical context of PROs to inform health system change approach

*Phase 1 (Inventory)* To inventory current UW Medicine PRO implementations, we identified needed data elements based on prior research [31] that describe health information technology implementation, including technology configuration and interface, user roles, workflow processes, and goals for data use (e.g., clinical practice, research, population health). To capture these elements, we distributed a semi-structured questionnaire to stakeholders across the UW health system with active involvement in the use of PROs for clinical care, and requested recommendations of additional stakeholders (i.e., snowball sampling) [32] who could help populate open inventory data elements. We distributed the survey through broad (i.e., health system-wide) and targeted (i.e., departmental) channels, and pursued additional stakeholders as available, including directed outreach to individuals who had made requests to IT services for new PRO measure builds for clinical care. The goal of the questionnaire was to inventory active PRO implementations, and therefore the team regularly reviewed questionnaire responses received with UW Medicine PRO stakeholders to ensure diverse representation and identify potentially missing respondents. PRO implementations that were exclusively research-based were excluded from the final inventory. Findings from this Phase cumulated in an inventory of PRO use across UW Medicine, and characteristics of PRO implementations including goals, clinical setting, mode of data collection (i.e., paper-based, electronic, interview), EHR integration, and PRO measures used.

*Phase 2 (Characterize)* To characterize common uses of PROs, we conducted semi-structured interviews with a convenience sample of stakeholders from eight PRO implementations selected from Phase 1. Interviews focused on understanding the context of PRO use. Participating stakeholders (*n* = 15) held diverse roles, including physicians (*n* = 8) representing surgery, pain medicine, oncology, neurology, psychology, general medicine, and infectious disease, nurses (*n* = 2), clinic support staff (*n* = 2), IT staff (*n* = 2), and healthcare administrators (*n* = 1). When possible, multiple stakeholders associated with a single PRO implementation were interviewed to understand diverse perspectives on PRO workflow and use. Interview questions (see Online Appendix A) expanded on the Phase 1 inventory to elicit stakeholders’ descriptions of how PROs were integrated into clinical workflows, technical and workflow design choices, and facilitators, barriers, and vision for the use and scale of PROs in practice.

We summarized notes from the interviews and performed qualitative content analysis [33] to characterize commonalities and differences across PRO implementations. We identified three use cases that reflect common patterns of PRO capture and reporting. These common use cases depict the core features (e.g., timing and frequency of data capture, use of PROs in clinical decision-making) of PRO implementations across clinical settings. We then used member checking [34] to verify insights and obtain additional feedback on proposed use cases.

*Phase 3 (Contextualize)* To enrich the development of system-wide PRO implementation strategy, we selected a single PRO measure as a practice scenario to apply across use cases. This allowed us to model the patterns of capture and reporting across diverse practice settings at UW Medicine, including similarities and differences in specialty and primary care. We chose the PHQ because it aligns with goals of the UW Medicine Depression Care Pathway and broader care transformation initiative. The PHQ-9 is a widely used and validated nine-item instrument for identification, diagnosis, and monitoring of depression [14, 35]. The PHQ-2 is an abbreviated version of the PHQ-9 utilized for depression screening. The PHQ has relevance for diverse stakeholders, including those involved in patient care across settings, population health, and quality improvement. Therefore the PHQ provided a robust measure to examine how to balance clinical setting nuances with health system standardization needs for PRO implementations [20]. We presented learnings to UW Medicine PRO stakeholders (UW PROs Governance and broader PRO learning community) at multiple points throughout our analysis for validation, feedback, and reflection.

## Results

### Phase 1: inventorying the current landscape of PRO implementations

We identified 14 PRO implementations spanning diverse clinical settings (Table 2), which reflect a breadth of PROs in use prior to formalized, system-wide PRO implementation efforts by UW Medicine. Goals for PRO capture ranged from screening to symptom monitoring to outcome assessment. Six implementations (43%) used only paper-based PRO data collection, five (36%) used only electronic data collection (e.g., patients directly entered PRO data through third-party platforms external to the EHR), and the remaining three (21%) used a mix of paper, electronic, and/or interviews with staff. EHR integration of PRO data was limited. Although 11 (79%) implementations stored PRO data in the EHR (e.g., PDF), only two (14%) integrated structured PRO data in the EHR as discrete values for real-time clinical decision support and reporting. The number of PRO measures collected in each setting ranged from 1 to 16; clinical settings using multiple PROs often reflected areas of care where disease or condition-specific measures were used, requiring the tailoring of PRO administration (e.g., adjusting the number and frequency of PRO measures) based on individual patient needs.

**Table 2:** Inventory of PRO implementations across the UW Medicine health system

Goal of PRO use	Clinical setting of the implementation	Mode(s) of data collection	Data storage approach in EHR	# of PRO measures
Health status screening	Cancer care	Electronic (web/app)	PDF	3
	HIV primary care	Electronic (tablet)	Unstructured text within EHR clinic note; Structured data in discrete EHR fields	13
Monitor symptoms	Multiple sclerosis	Interview with clinical staff; paper	Unstructured text within EHR clinic note	3
	Sports medicine	Paper	PDF	8
	Head and neck cancer	Paper	PDF	3
	Prostate cancer	Paper	PDF	2
	Pain management	Electronic (web/app); paper	PDF	16
	Pediatric mental health	Electronic (web)	Not stored in EHR	*Varies by diagnosis
	Post-surgical wounds	Electronic (web/app)	Not stored in EHR	1
	Headache management	Electronic (web)	Unstructured text within EHR clinic note	6
Assess outcomes	Urologic surgery	Paper	PDF	8
	Orthopedic surgery	Paper	PDF	6
	Reconstructive surgery	Paper	Not stored in EHR	1
	Spine/total joint surgery	Electronic (patient portal); interview with clinical staff	Structured data in discrete EHR fields	2

The inventory of PROs provides a detailed characterization of individual PRO implementations, and an opportunity to assess the potential alignment and overlap of PROs when scaled across the system. Among the 14 implementations inventoried, there were over 73 PRO measures in use, over half of which (53%) captured condition- or population-specific outcomes, such as the *Disabilities of the Arm, Shoulder and Hand* (DASH) scale. The remaining (47%) captured shared clinical domains (e.g., quality of life, depression, pain) with applicability to multiple care teams across the health system (see Table 3). As many as five different PRO measures were used to capture each shared domain, and there were nine examples of duplicate measures, meaning identical measures were captured in multiple clinical settings without the ability to transfer data between settings.

**Table 3:** Examples of PRO measures in shared domains available for use across health system

Shared domain	PRO measures available outside EHR (*e.g., paper, third-party platform*)	PRO measures available within EHR (*e.g., patient questionnaires, flowsheets*)
Depression	PHQ2, PHQ4, PHQ9	PHQ4, PHQ9, Major depressive inventory, Geriatric Depression scale
Anxiety	Generalized anxiety disorder (GAD) 7, PHQ4	Generalized anxiety disorder (GAD) 7, PHQ4
Pain	Numeric rating scale, Visual analog scale, Pain severity, Pain diagram	(None available)
Quality of life	PROMIS 10, PROMIS 29, SF-12, EQ 5D, Functional Assessment of Cancer Therapy-General (FACT-G)	PROMIS 10, EQ 5D

### Phase 2: characterizing common use cases for PROs in clinical care

The goal of Phase 2 was to provide more context around how PROs were currently used in clinical practice and characterize the core attributes of PRO tools and workflow that would support successful use at the health system level. Interviewees described experiences and learnings that facilitated better PRO data collection and use, including engaging all staff members in the PRO data collection process and providing adequate training for providers on how to integrate PRO data into clinical decision-making. Interviewees also described persisting barriers to PRO scale and spread, highlighting the need for significant technology customization within PRO tools, challenges related to EMR integration (e.g., where should PRO data live), and the resulting negative impact on workflow alignment and efficiency. Overall, three qualitative themes emerged from interviews that characterize the nuances of PRO data collection and reporting practices: (1) *value* (i.e., the importance of patient-reported data to complement clinical data and facilitate patient-centered care), (2) *actionability* (i.e., the importance of measures that align with clinical decision-making), and (3) *technology alignment* (i.e., the need for technology to fit with current workflow and support real-time PRO data use). Stakeholders highlighted that the technological configurations of PRO data collection and reporting tools were largely driven by the role of PRO data in clinical decision-making at the point-of-care. These insights informed the development of three use cases that illustrate the role of PROs across the healthcare system: (1) *Preventive care,* (2) *Chronic/Specialty care*, and (3) *Interventional/Surgical care.*

### Phase 3: contextualizing application of use cases in care delivery

**Fig. 1:**
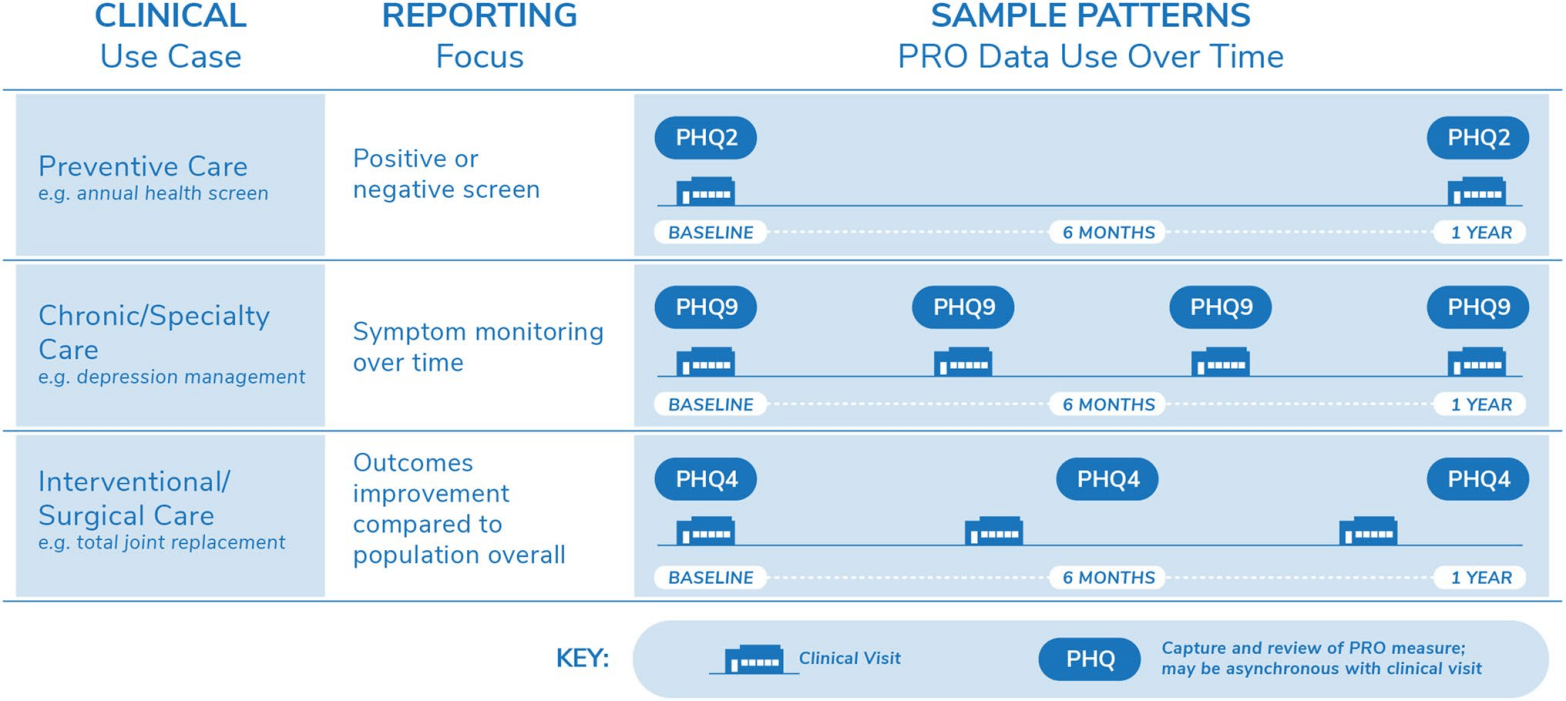
Application of the PHQ across three common PRO use cases: Evaluating depression in preventive, chronic/specialty, and interventional/surgical care

Based on findings from Phase 1 and 2, depression emerged as a common health domain for PRO assessment across the three use cases. In preventive care, providers actively screen for depression as part of annual wellness visits. In specialty care, depression may be the primary focus of treatment (behavioral health) or a co-existing condition that may be exacerbated with changes to treatments or natural progressions in health (i.e., multiple sclerosis, heart failure). In surgical care, depression is often a risk factor for poor outcomes and thus screening for the condition may be included in perioperative planning. While the importance of depression is shared across the different use cases, the value, actionability, and alignment with technology varied as the clinical need moved from preventive care towards specialty care. Figure 1 presents how we leveraged the PHQ to consider the nuances of the three use cases, and the scenarios that follow detail how the use cases illuminate strategies for system-wide implementation.

In *preventive care*, PROs like the PHQ support screening, diagnosis, and decisions regarding depression treatment or referrals to specialty services. Compared to other use cases, the preventive care timing is the least frequent (e.g., annually), and therefore PRO data collection often needs to incorporate many measures, influenced by both clinical needs and payer-driven requirements. For preventive care, brief, easy to administer screeners like the PHQ-2 are ideal as they provide actionable data with minimal patient burden. Scoring of PRO assessments often focuses on a straightforward dichotomy (i.e., positive/negative, high/low risk), resulting in minimal PRO reporting with a primary goal of alerting clinical and population health teams to the need for collection of additional information and/or coordination of care.

In *chronic/specialty care,* PROs support symptom management for monitoring chronic health conditions, such as management of active depression by primary care or behavioral health care teams. Compared with preventive care, fewer PRO measures may be used, with more frequent data collection time points often corresponding with regularly scheduled appointments. Clinical teams are more likely to individualize the administration of PRO measures, tailoring and adjusting PRO assessment (i.e., content, frequency) to each patient’s health status. Additionally, the need for longitudinal visualizations emerges, such as reporting tools that combine PRO and clinical data in customized views to track individual trajectories of symptoms and treatment response. When the PHQ-9 is used in chronic/specialty care, it is critical to obtain complete assessments to generate a cumulative score of the items at every visit as well as explore responses to individual items that report specific symptoms. PHQ-9 scores can then inform diagnosis and disease progression, track symptom burden, monitor impacts of treatment changes (e.g., medication changes), and evaluate care intensity needs, including recovery or remission.

In *interventional/surgical care*, PROs are often used to compare outcomes for specific populations over a defined period of time, such as one-year function and pain outcomes for patients undergoing total joint replacement surgery. The workflow for PRO collection becomes increasingly complex as factors, such as nuanced population identification (e.g., multiple inclusion or exclusion criteria) and discrete time windows for data collection, are applied. PRO reporting in these implementations focuses on visualizations that filter and compare PRO data by demographic or clinical patient factors. PRO data collection intervals are based around the intervention being monitored, and may be less aligned with visits where clinical teams have the opportunity to collect PRO data from patients directly. Another distinction is that in interventional/surgical care, PRO measures such as the PHQ-2 are used not to inform a patient’s depression care needs, but to assess the fitness of patients for major interventions. However, this introduces the need to establish support structures to ensure clinical teams can respond to depression screening results with appropriate care referrals. At the population level, such data serve to describe potential moderators of clinical outcomes in aggregate analyses.

Distinguishing the characteristics of these common use cases is valuable not only to inform the successful implementation of PROs, but also to illustrate challenges health systems face when scaling PROs system-wide. In the examples provided, a single measure of depression would need to address the workflow and reporting needs of three different use cases. Review of these three use cases with our health system stakeholders identified the need to govern the selection of PRO measures, such as the use of the PHQ for depression, so as to balance the needs across use cases and support consistent measurement. Understanding the application of common data needs across different settings of care can therefore support efficient design of system-wide data collection and reporting tools.

## Discussion

Health systems increasingly aim to integrate the patient voice into clinical care through routine capture of PROs and other forms of patient-generated health data. As the use of these new forms of data continues to increase, so does the need for health systems to understand how to respond in ways that achieve the goals for practice transformation while thoughtfully managing limited resources. A critical element of this effort will be system-wide governance of how PROs are prioritized, designed, and managed in practice [36]. While the role of formal governance is still an emerging practice in many areas of health system management, the goals of governance work often center around engaging stakeholders with diverse expertise, establishing shared goals, aligning incentives and resources to facilitate work, and building infrastructure to advance practice through standardization and continuous improvement [37, 38]. Learnings from our work with UW Medicine, consistent with other systems’ experience [8, 25, 39–42], demonstrate the invaluable role governance could play in supporting health system use of PROs. In particular, governance for PRO implementation can ensure that PRO measurement selection, technology configurations, and workflow design are driven by stakeholder priorities and health system capabilities.

The three use cases presented provide a nuanced example of how governance structures are needed when PROs are scaled across healthcare organizations in order to enhance efficiencies and promote adoption [43]. Prior work, such as ISOQOL’s *User’s Guide to Implementing Patient*-*Reported Outcomes Assessment in Clinical Practice* [42] and PCORI’s *User’s Guide to Integrating Patient*-*Reported Outcomes in Electronic Health Records* [25], have emphasized the need for thoughtful measurement selection that complements clinical decision-making. Our findings advance these recommendations by demonstrating the cascading impacts on health system resources and the experiences of patients and care teams when a system-wide measurement strategy is not defined. The PHQ, for example, was one of several PRO measures used for depression measurement at UW Medicine, highlighting the shared interest in assessing depression across care settings. Yet other PRO measures had more localized uses within a single clinic. In contrast to how measurement systems such as PROMIS [44] and measurement initiatives such as the International Consortium for Health Outcomes Measurement (ICHOM) [45] have conceptualized the organization of PRO measures around clinical disease areas, some health systems may instead need to organize and prioritize PRO measures around patterns of use across areas of clinical care. Strategies that are informed by implementation, such as the standardization of PRO measurement for common domains [11, 15], may better enable health systems to address the inefficiencies that can arise without intentional governance of resources [8]. Through an approach that balances and governs clinical commonality, health systems can better scale and sustain infrastructure for PRO implementation while reducing measurement burden on patients.

This study demonstrates the value of characterizing the diversity of PRO implementations across large health systems to inform practice transformation initiatives. The three use cases—*preventive care, chronic/specialty care*, and *interventional/surgical care*—provide useful insight into reporting needs for different stakeholders within the healthcare system. The need for depression measurement with the PHQ remains important across clinical contexts, for quality improvement, population health management, and regulatory and contractual reporting [36, 46, 47]. However, there are limitations to consider. First, our work focused on the needs of clinical teams and health systems, and does not reflect the patient perspective, although such work is underway. Second, we used snowball sampling for conducting our cataloging exercise. We allowed contacts to forward on the link to the questionnaire rather that requesting contact information to send the questionnaire directly and thus our ability to fully understand breadth of outreach and response rate is limited. Third, while we sought to obtain a robust view of PRO collection in clinical practice, we anticipate that not all PRO measures in current use were captured, especially those captured only by a single provider, not a clinic-wide implementation. This reflects the lack of formal integration of PRO data within clinical workflows and established channels for assessing such practice in health systems. Lastly, as with all action research, the extent to which findings can be generalized may be best assessed by their application in other contexts [26]. Future work should further refine organizing frameworks, such as our use cases, that can guide healthcare systems in the design of system-wide PRO governance and implementation strategies.

Practice transformation efforts increasingly require integration of the patient voice into clinical care, often through the use of PROs. Our work adds to the growing evidence on system-wide PRO implementation by demonstrating how common patterns and domains of use warrant the need for PRO governance to optimize the design of PRO tools. Learnings from our work highlight the importance of proactively considering how PROs will be used across the layers of healthcare organizations in order to bridge health system goals for clinical quality, sustainability, practice transformation, and the drive towards patient-centered care.

## Electronic supplementary material

Below is the link to the electronic supplementary material.

Supplementary material 1 (DOCX 15 kb)
