# Supplementary material for: Capturing the patient voice: implementing patient-reported outcomes across the health system
Source: Qual Life Res. 2019 Oct 12;29(2):347–55. doi: 10.1007/s11136-019-02320-8 (PMC6995985; doi:10.1007/s11136-019-02320-8)
Supplement: Supplementary file 1 — Supplementary material 1 (DOCX 15 kb) [file 11136_2019_2320_MOESM1_ESM.docx]

**Appendix A: Semi-structured interview guide for PRO Case Study Explorations**

1. Do you currently collect patient reported outcomes (PROs) data?
2. What area of care or patient population do you collect patient reported outcomes for?
3. What is the primary purpose of your PRO data collection?
4. What type of PRO data do you collect directly from patients (e.g. what measures do you collect)?
5. Do you collect additional types of data (e.g. clinical history, patient-generated data) alongside the PRO data?
6. Can you describe the tool or system you use to collect and/or store your PRO data?
7. When do you collect PROs? For example, is your PRO data collection driven by clinical visits, or clinical events or timepoints?
8. What method do you use to collect PRO data from patients?
9. What formats do you use for patient outreach to complete PROs?
10. Where do patients normally complete the PRO measures?
11. What is the relationship between your PRO system and the electronic medical record?
12. What data visualization capabilities does your PRO system have?
13. How do you view your PRO data?
14. What roles (clinical, administrative) can view the PRO data?
15. Who are the PRO visualizations designed for?
16. Who do you share your PRO data reports with?
17. What have been the barriers or challenges to your use of PROs?
18. What is your vision for how PROs should integrate with clinical care? What improvements to your current system or tools would you like to see?
19. Is there anything else you’d like to share about your experience with PROs?
20. Is there anyone else you recommend we talk to about their use of PROs?
